# The Detection and Bioinformatic Analysis of Alternative 3^′^ UTR Isoforms as Potential Cancer Biomarkers

**DOI:** 10.3390/ijms22105322

**Published:** 2021-05-18

**Authors:** Nitika Kandhari, Calvin A. Kraupner-Taylor, Paul F. Harrison, David R. Powell, Traude H. Beilharz

**Affiliations:** 1 Development and Stem Cells Program, Department of Biochemistry and Molecular Biology, Monash Biomedicine Discovery Institute, Monash University, Melbourne, VIC 3800, Australia; nitika.kandhari@monash.edu (N.K.); Calvin.Kraupner-Taylor1@monash.edu (C.A.K.-T.); paul.harrison@monash.edu (P.F.H.); 2 Monash Bioinformatics Platform, Monash University, Melbourne, VIC 3800, Australia; David.Powell@monash.edu

**Keywords:** alternative polyadenylation, bioinformatics, 3′ focused RNA-seq, scRNA-seq, cancer biomarkers

## Abstract

Alternative transcript cleavage and polyadenylation is linked to cancer cell transformation, proliferation and outcome. This has led researchers to develop methods to detect and bioinformatically analyse alternative polyadenylation as potential cancer biomarkers. If incorporated into standard prognostic measures such as gene expression and clinical parameters, these could advance cancer prognostic testing and possibly guide therapy. In this review, we focus on the existing methodologies, both experimental and computational, that have been applied to support the use of alternative polyadenylation as cancer biomarkers.

## 1. Introduction

Eukaryotic messenger RNA (mRNA) undergoes a highly regulated process of maturation before nuclear export and protein translation. This involves 5${}^{\prime }$ end capping, RNA-splicing and 3${}^{\prime }$ end cleavage and polyadenylation. Initially thought to be a static housekeeping function, mRNA 3${}^{\prime }$ end formation has emerged as a major modulator of gene expression with implications in multiple disease settings [1,2].

Alternative polyadenylation (APA) is a regulatory mechanism that allows the production of coding and regulatory transcript isoforms from a single gene [3,4,5,6]. This occurs due to the presence of alternative adenylation sites in the genome and leads to significant transcriptome diversity. Nearly 70% of mammalian genes harbour multiple cleavage and polyadenylation sites i.e., poly(A) sites [7,8,9,10]. These sites can cause differential expression of mRNA transcripts by influencing their nuclear export, stability, subcellular localization, interaction with microRNAs, RNA binding proteins (RBPs), long non-coding RNAs (lncRNAs) and translation efficiency [11,12,13,14,15].

**Figure 1:**
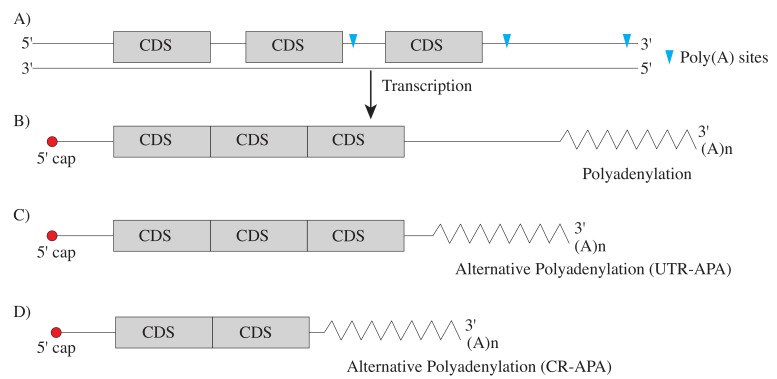
**Alternative polyadenylation:** (**A**) The schematic shows the 5${}^{\prime }$ end, coding sequences (grey boxes), 3${}^{\prime }$UnTranslated Regions (3${}^{\prime }$ UTRs) and polyadenylation sites (blue arrows) in DNA. (**B**) Polyadenylation is the enzymatic extension of ∼200 Adenosine residues to the nascent mRNA, in this case the distal polyadenylation site was used. (**B**,**C**) In 3${}^{\prime }$ UTR-APA, choice of the proximal cleavage and polyadenylation results in an mRNA with the same protein-coding potential but different 3${}^{\prime }$ UTR length. (**D**) When a poly(A) signal is recognised in the intronic region, protein isoforms with distinct Carboxy-termini are generated in a process termed as CR-APA.

**Figure 2:**
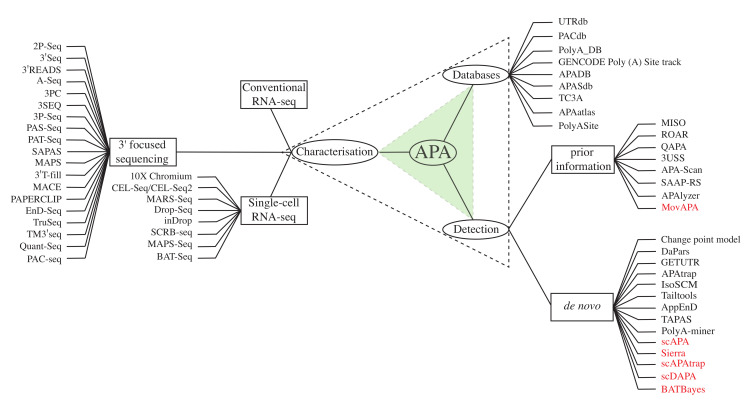
The triad of APA attributes: This review focuses on three attributes of genome-wide APA i.e., characterisation, detection and curation of APA databases. Currently, conventional RNA-seq, 3${}^{\prime }$ focused seq and single-cell RNA-seq are the main methods for APA characterisation. APA databases hold information relating to APAs and 3${}^{\prime }$ UTRs collated from a wide array of inputs. Detection requires bioinformatic methods for statistical ranking. These methods are classified based on prior knowledge from the databases or determined *de novo*.The bioinformatic methods for single-cell data analysis are shown in red.

Two major types of APA events are described here; splicing-APA where protein sequence is changed, and tandem APA where only the extent of non-coding, regulatory information is altered (Figure 1). In the case of splicing-APA, the alternative poly(A) sites reside in introns of the coding sequences, generating protein isoforms with distinct Carboxy-termini. Such APAs are called coding region-APA (CR-APA) [16,17,18]. In the case of tandem APA, the poly(A) sites reside in the 3${}^{\prime }$ UTRs resulting in transcript isoforms with invariant protein-coding sequence but 3${}^{\prime }$ UTRs of different lengths. Such APAs are called UTR-APA [16,17,18]. In this review, we discuss the implications of APA and investigate the existing experimental and bioinformatic methods for detection, quantification and identification (Figure 2). Finally, the emerging role of APA signatures as cancer biomarkers will be explored.

## 2. Implications of Alternative Polyadenylation

Since the discovery of APA in immunoglobulin M (IgM) and dihydrofolate reductase (DHFR) genes in 1980 [19,20], it has become clear that APA is the norm rather than the exception. At least 70% of human genes are subject to APA, and 3${}^{\prime }$ UTR changes are often associated with physiological conditions including diseases such as cancer, immune dysfunction, congenital heart disease and dysplasia [21]. Where genes have the capacity to switch, short 3${}^{\prime }$ UTRs generally associate with undifferentiated proliferative cells (e.g., stem cells) whereas the longer 3${}^{\prime }$ UTR isoforms are favoured in differentiated tissues [22,23,24]. It has been suggested that the majority of APA genes switch to short mRNA isoforms in tumour cells [23,24,25]. Where there is an option to switch, mRNAs with longer 3${}^{\prime }$ UTRs can cause reduced protein expression as a result of increased regulatory capacity. Whereas, increased stability and translation of short 3${}^{\prime }$ UTR isoforms are some of the key functional consequences suggested for APA; for example, due to loss of microRNA-mediated repression [22,23]. APA-mediated evasion from microRNA repression can generate stable oncogenic mRNA isoforms with shorter 3${}^{\prime }$ UTRs causing oncogenic activation [23]. It is important to note, however, that there are many exceptions to this trend. For example, the long-3${}^{\prime }$ UTR isoform of the tumour suppressor PTEN is the more stable isoform and accounts for the bulk of its role in PI3K/AKT/mTOR signalling [26]. Albeit, the net consequence of 3${}^{\prime }$ UTR shortening of PTEN still promotes tumour growth through reduced tumour suppressive activity.

Dynamic APA regulation has been reported in different healthy tissue types [27] in cellular proliferation, differentiation and development; in cancer cell transformation, and phenotypic response to extracellular stimuli [5,23,28,29,30,31,32,33,34,35,36,37]. For example, selection of a proximal poly(A) site resulting in 3${}^{\prime }$ UTR shortening has been shown to associate with multiple cancers [25,38,39,40,41]. APA-mediated changes by CR-APA can diversify protein function. For example, a switch from proximal to distal APA in the IgM gene, results in a switch from a secreted to membrane-bound form of the antibody [42]. mRNAs with longer 3${}^{\prime }$ UTRs can be subject to increased regulation and reduced protein expression. This is due to the inclusion of regulatory sequences such AU-rich and GU-rich sequences, RBP and miRNA target sites all of which can negatively impact mRNA stability and/or translation efficiency [5]. As a result, shorter mRNA isoforms can escape regulation by loss of such sites leading to increased RNA stability and enhanced protein expression [23,33]. In addition to regulation in mRNA and it’s encoded protein, seminal work by Berkovits and Mayr (2015) shows that 3${}^{\prime }$ UTRs can serve as a physical scaffold for ternary complex formation [13]. Alternative polyadenylation in long non-coding RNA has also been described and plays a role in tumorigenesis [43].

## 3. Next-Generation Sequencing Based Techniques for Characterisation of APA

Global profiling of APA first became possible through accumulation of expressed sequence data in public databases and the development of high-content microarray. Bioinformatic analysis of expressed sequence tags (ESTs) and microarray studies helped detect many APA events in the late 90 s [20,33,36,44,45,46]. Soon however, RNA sequencing (RNA-seq), became the major method for transcription profiling [47]. With RNA-seq it became possible to study the complete transcriptome by massively parallel short-read sequencing of cDNA libraries, allowing differential analysis of the gene expression between samples. Combined with biostatistics, this approach identified genes, and alternative isoforms of genes [47,48]. One of the drawbacks of bulk full-length RNA-seq, however, is an overall loss of read coverage of 5${}^{\prime }$ and 3${}^{\prime }$ ends of genes making it unreliable for detection of alternative transcriptional start-sites and APA [49]. Moreover, for many applications where only differential expression was required, sequencing the full-length transcriptome was unnecessary and costly. This motivated researchers to develop both 5${}^{\prime }$ and 3${}^{\prime }$ focused sequencing methods to sequence the specific transcriptomic regions of interest.

### 3.1. 3${}^{\prime }$ focused RNA-seq Methods for APA Characterisation

Early studies for APA identification used Direct RNA-sequencing (DRS) [50] with the Helicos platform, now replaced by Oxford Nanopore and PacBio (Table 1). These provide a quantitative view of APAs genome-wide, but are expensive and relatively low throughput. However, given that only the reads mapped to the 3${}^{\prime }$ ends of mRNA are necessary for APA detection, a more pragmatic approach was to sequence only the mRNA 3${}^{\prime }$ ends based on classic 3${}^{\prime }$ RACE methods [51]. Most 3${}^{\prime }$ focused methods enrich RNA carrying a poly(A) tail and include a variety of molecular biology methods to generate a library suitable for next generation sequencing [17]. The resulting sequencing data are bioinformatically analysed for identification of poly(A) sites and quantification of their differential usage. Current commercial and bespoke approaches to transcriptome-wide characterisation of APA are listed in Table 1.

In general, 3${}^{\prime }$ focused methods use oligo(dT) primers to target the poly(A) tail and thereby enrich sequencing of Poly(A)+ mRNAs. The steps that result in inclusion of sequencing adaptors, unique molecular identifiers (UMIs), size selection and library amplification are often varied between approaches. However, an RNA fragmentation step or other means to limit sequencing libraries to the region directly upstream of poly(A) sites is always included. Methods that use oligo(dT) primers bias away from ribosomal RNA and other non-poly(A) RNA during reverse transcription. Albeit, rRNA decay intermediates carry poly(A)-tails and these can be abundantly detected. The use of oligo(dT) primers can cause significant mis-priming at internal A-rich regions leading to false poly(A) site identification. This can be addressed *in silico* by eliminating the putative poly(A) residues in A-rich regions [37,72]. Approaches that use 3${}^{\prime }$ end ligation are less prone to mis-priming than those where cDNA synthesis is driven from annealed oligo(dT) primers. Both *in silico* and *in vitro* strategies have thus been developed to avoid the problem of internal priming [5,73]. PAPERCLIP, which uses immune-purification of the poly(A)-binding protein is an alternative method for detection of mRNA 3${}^{\prime }$ ends [65,74]. While the methods discussed here focus on APA and 3${}^{\prime }$ UTR isoforms, a subgroup of 3${}^{\prime }$ focused sequencing methods additionally identify poly(A) tail length changes [54,56,57,58]. Finally, although direct RNA sequencing is currently the least affordable technology, it is the only method that can integrate APA with other mRNA processing events, such as alternative transcriptional start-site and splice sites.

### 3.2. Single-Cell Methods for mRNA 3${}^{\prime }$ End Sequencing

High content research is experiencing a dramatic shift towards single-cell methods. Single-cell RNA-seq (scRNA-seq) allows transcriptome-wide analyses of gene expression in individual cells with high resolution [75] for discovery of novel cell types and their developmental trajectories [76,77,78]. The single cell methods include early cell-barcoding of samples which allows individual samples to be pooled and processed as a single sample. Early pooling (or early multiplexing) of samples significantly reduces the costs and increases sequencing-throughput [69]. Another interesting feature of single-cell RNA-seq methods is the use of UMIs, which allows detection of PCR duplicates while reporting the unique transcript counts and thus, removes PCR amplification bias [79,80]. Most scRNA-seq methods use 3${}^{\prime }$ tag-based approach to generate reads enriched at 3${}^{\prime }$ ends of mRNA similar to the approaches described above (Table 1). Several laboratories have already turned to scRNA-seq to study complex APA regulatory patterns in tissues and organs [10,81,82,83].

There are two major methods of scRNA-seq library generation that allow APA detection: Micro-well based methods and Microfluidic droplet-based methods. In microwell-based methods, cells are separated into microwells for barcode allocation and their transcriptome is reverse-transcribed; whereas in microfluidic droplet-based methods, individual cells are separated using nanolitre-sized droplets containing reagents for UMI and cDNA synthesis [84,85]. Each cell is lysed and mRNA 3${}^{\prime }$ ends are annealed to primers containing UMI followed by RT reaction to generate the first cDNA strand. cDNAs are pooled for library amplification and sequencing. The information from individual cells is distinguished *in silico* based on the UMIs. The single cell approaches that allow detection of APA are listed in Table 2.

We have broadly classified the APA characterisation techniques into three categories: conventional RNA-seq, 3${}^{\prime }$ focused RNA-seq and scRNA-seq methods (Figure 2). In the next sections, the bioinformatic tools available for 3${}^{\prime }$ UTR detection and databases to store curated forms of this information are described.

## 4. Bioinformatic Methods for Detection of Poly(A) Sites

**Figure 3:**
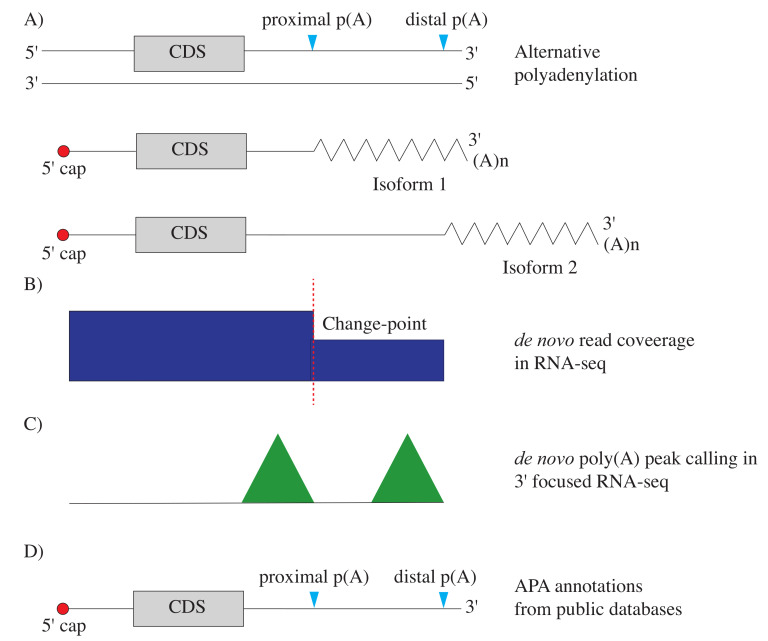
**Detection of poly(A) sites:** (**A**) Two polyadenylation sites, proximal and distal, result in expression of two isoforms. (**B**–**D**) Methods to determine the location of poly(A) sites: (**B**) *de novo* method to identify change-points in read-coverage of RNA-seq data. (**C**) *de novo* method to identify poly(A) peaks in 3${}^{\prime }$ focused RNA-seq data. (**D**) combining read-coverage data with poly(A) site coordinates from APA databases.

Bioinformaticians have sought to extract poly(A) site usage information from sequencing data, either using inference from read coverage in conventional RNA-seq or by quantitating read coverage data from the 3${}^{\prime }$ focused methods (Figure 3). Some of these methods use known annotations from curated databases, whereas others identify peaks *de novo*. In this section, the existing bioinformatic tools for the detection of poly(A) sites from the sequencing data are discussed.

### 4.1. Databases for 3${}^{\prime }$ UTR and APA Storage and Retrieval

The rapid accumulation of high-throughput data paved the way for investigation of RNA isoforms in a variety of physiological and pathological conditions [47,48]. RNA-seq emerged as a reliable tool to study transcriptome diversity due to its quantitative detection of alternative transcriptional start-site, splicing and APA events at nucleotide resolution. Public databases were created to store experimentally determined poly(A) sites and 3${}^{\prime }$ UTR variants. In this section, we review the existing databases that catalogue the 3${}^{\prime }$ UTRs in various organisms [27,93,94,95,96,97,98,99].

The primary data were collected from EMBL annotation records (UTRdb), transcript genome alignments in cDNA/ESTs (PACdb, PolyA_DB3, PolyA site track) inferred from RNA-seq (TC3A, APAatlas) or curated from 3${}^{\prime }$ focused RNA-seq (APADB, APASdb, PolyASite) (Table 3). Unfortunately, a number of useful resources have not been maintained (e.g, PACdb [95], APASdb [96] and TC3A [99]) and/or have been incorporated into updated resources. This leaves two main approaches for determination of global APA. (1) The bioinformatic extraction from consortium resources such as the Ensembl database, or more specifically GENCODE PolyA site track [100,101] which holds high-quality annotations for coding and non-coding regions and pseudogenes in the human genome. Or, (2) The use of specifically curated APA databases. The latter are collated from either direct 3${}^{\prime }$ focused sequencing or by inference from RNA-seq. For example, APADB [97] reports poly(A) sites for coding and non-coding transcripts in human, mouse and chicken and reports the loss of predicted miRNA binding sites from MACE-seq data. Whereas, PolyASite 2.0 [98] contains the most up to date curation from a multitude of 3${}^{\prime }$ focused RNA-seq methods, re-analysed by protocol-specific data pre-processing steps for consistency in APA mining. Gene tracks can be downloaded for genome browser exploration. PolyA_DB3 [94] provides information about the genomic locations of poly(A) sites and the surrounding *cis* elements and a comparison of polyadenylation configuration between human and mouse orthologs. UTRdb [93] curates 5${}^{\prime }$ and 3${}^{\prime }$ UTR sequences and provides information about genome localisation and regulatory elements. It is integrated with UTRsite [93] which is a collection of experimentally validated functional regulatory motifs in 5${}^{\prime }$ and 3${}^{\prime }$ UTRs crosslinked with their protein partners. This integration allows users to retrieve data based on genomic coordinates and/or genes associated with encoded proteins using GO terms, PFAM domains, etc.

There is, however, still a relatively low availability of 3${}^{\prime }$ focused RNA-seq data. Many cell, tissue and disease types are still missing, limiting the scope of these databases. To overcome this limitation, APAatlas [27] provides a resource database of APA inferred from RNA-seq data in the Genotype-Tissue Expression (GTEx) project [102] using the DaPars [25] bioinformatic approach (see Section 4.2.2). A similar approach was recently used to mine RNA-seq from The Cancer Genome Atlas (TCGA) [103] where the inferred APA genes are provided in TC3A [99].

The annotation from these databases are useful for visualisation and interpretation of APA genome browsers such as the UCSC Genome Browser [104] or the Integrated Genome Browser [105]. Moreover, many tools for APA detection and quantification depend on database annotations to guide bioinformatic analysis as discussed in the section below.

### 4.2. Bioinformatic Methods for APA Detection and Quantification

The increasing interest in 3${}^{\prime }$ UTR dynamics, and the growth of associated technologies required design of bioinformatic tools. Multiple approaches were designed to infer APA from conventional RNA-seq, as well as tools to extract it from 3${}^{\prime }$ focused RNA-seq methods. Some APA detection methods rely on prior knowledge, while others involve the *de novo* detection of poly(A) sites.

#### 4.2.1. APA Detection in RNA-seq Data Based on Prior APA Information

The section below provides a brief overview of the bioinformatic methods available for inference of APA from read-coverage in RNA-seq data, where known APA sites are used to guide analysis. The use of data-base derived APA information improves the accuracy of *in silico* APA detection.

Mixture of ISOforms (**MISO**) [107] was the first reported tool for detecting previously annotated 3${}^{\prime }$ UTR isoforms, using a probabilistic framework to quantify alternative splicing (AS) and alternative polyadenylation. It identifies the differentially regulated AS/APA isoforms from the expression levels and delivers the probability of the origin of a read from a particular transcript isoform.

Ratio Of ARatio (**ROAR**) [108] is an R-based program that identifies differential APA site usage in RNA-seq. The algorithm defines two distinct 3${}^{\prime }$ UTRs in a gene, guided by APA databases, one which is shared by both the short and long 3${}^{\prime }$ UTR isoform and the other which is present only in the long 3${}^{\prime }$ UTR isoform. It scans the read-coverage for these two 3${}^{\prime }$ UTR isoforms and computes the expression ratio (m/M) of reads falling in the two regions. To compare between conditions, the ratio of two isoform-expression ratios (m/M) is computed in different samples and is called the Ratio Of A Ratio. This ratio represents the tendency of expression of a short isoform or a long isoform in a given condition. A roar >1 indicates higher levels of short isoform (a roar <1 indicates higher levels of long isoform) in the first condition. This method derives APA annotations from APASdb and PolyA_DB2 [72].

Quantification of APA (**QAPA**) [9] uncovers APA from RNA-seq data by retrieval of 3${}^{\prime }$ UTR annotations in GENCODE Poly (A) site track [101] and PolyASite 2.0 [98] and use these to construct an expanded reference library of annotated poly(A) sites and 3${}^{\prime }$ UTR sequences. The sequences in this library are used to measure expression from RNA-seq data and estimate relative abundance of alternative 3${}^{\prime }$ UTR isoforms. The method directly estimates the absolute alternative 3${}^{\prime }$ UTR isoform expression from protein-coding genes. Then it computes the relative expression of each 3${}^{\prime }$ UTR isoform among all isoforms to assess APA.

3${}^{\prime }$UTR Sequence Seeker (**3USS**) [109] is a web-server that analyses the transcript assembly file and automatically identifies transcripts with alternative 3${}^{\prime }$ UTRs with respect to the reference genome of choice. The 3${}^{\prime }$ UTRs are identified as the regions located immediately downstream of the stop-codon. These are then compared with previously annotated 3${}^{\prime }$ UTRs in public databases, iGenomes (https://sapac.support.illumina.com/sequencing/sequencing_software/igenome.html) and GENCODE [100,101] to identify novel 3${}^{\prime }$ UTRs and to detect length differences amongst existing and putative novel 3${}^{\prime }$ UTRs. It provides the nucleotide sequence of the 3${}^{\prime }$ UTR isoform along with their genomic coordinates and the UTR length differences.

**APA-Scan** [110] identifies genome-wide UTR-APA events by utilizing the predicted or experimentally verified poly(A) signals as reference for poly(A) sites estimating the 3${}^{\prime }$ UTR read coverage from both aligned RNA-seq and 3${}^{\prime }$ end-seq data to identify potential poly(A) sites. Then it pools all the aligned reads to identify peaks and cleavage sites in 3${}^{\prime }$ UTRs which are considered as potential poly(A) sites. It performs a $\chi^{2}$-test on the experimentally determined or predicted cleavage site in the 3${}^{\prime }$ UTR to compare APA between samples.

Significance Analysis of Alternative Polyadenylation using RNA-Seq (**SAAP-RS**) [111] uses RNA-seq samples from bulk, single cell and 3${}^{\prime }$ focused (e.g., 3${}^{\prime }$ READS+ [10]) approaches to identify APA events. The method calculates RNA-seq read counts upstream (UP) and downstream (DN) of every poly(A) site identified from PolyA_DB3 database and performs a statistical test to derive a p-value to compare the read distribution in UP and DN regions between two samples. The relative expression difference (RED) of the APA isoforms is used to identify genes with significantly altered 3${}^{\prime }$ UTR lengths between cell types.

**APAlyzer** [112] is a Bioconductor package for identification of APAs in 3${}^{\prime }$ UTR and intronic regions by calculating the RNA-seq read density (RD) after splitting the transcript 3${}^{\prime }$ end regions based on the annotations derived from PolyA_DB3.

Due to their dependence on incomplete information of poly(A) sites, MISO, ROAR, 3USS and APAlyzer may fail to detect uncharacterised APAs.

#### 4.2.2. *de novo* APA Detection in RNA-seq Data

These are the bioinformatic methods that detect 3${}^{\prime }$ UTR switching events in RNA-seq data without relying on prior knowledge. The methods use a variety of approaches, but a majority of tools scan the read-coverage to detect “change-points”. A change point is a critical point that marks the shift or transition in the depth of read-coverage (Figure 3). The presence of more than one 3${}^{\prime }$ UTR isoform creates a “step-down” inferred as the change points that define the APA boundaries.

Dynamic analysis of Alternative PolyAdenylation from RNA-Seq (**DaPars**) [25] performs *de novo* identification of APA in RNA-seq experiments. The method scans the read-coverage and identifies a distal poly(A) site present at the end-point of the longest 3${}^{\prime }$ UTR among samples. It then seeks a model providing the best least-squares fit of the read-coverage along the gene up to the identified distal site. This model consists of the location of a proximal poly(A) site and the expression levels of the short and long isoforms in each of the two conditions. This best model provides both the location of a proximal site and the information required to calculate the "Percentage of Distal polyA site Usage Index" (PDUI) for each condition.

Tool for Alternative Polyadenylation site AnalysiS (**TAPAS**) [113] deals with more than two APA sites in genes as well as 3${}^{\prime }$ UTRs with intronic regions. The tool is based on multiple change point inference model for finding change points in time series data, but applies more stringent filtration techniques to discard false APA sites. The method is extended to identify APA sites that are differentially expressed across samples to infer genes that undergo 3${}^{\prime }$ UTRs shortening/lengthening.

Global Estimation of The 3${}^{\prime }$UTR landscape based on RNA-seq (**GETUTR**) [114] is a Python-based method that uses RefSeq gene annotations to provides a landscape of 3${}^{\prime }$ UTR and finds poly(A) sites by smoothing read-coverage to flatten the erroneous variations in the RNA-seq signal. The smoothing technique may generate many false poly(A) sites.

Isoform Structural Change Model (**IsoSCM**) [115] is a standalone transcript assembly tool that annotates mRNA 3${}^{\prime }$ ends based on multiple change-point analysis to generate complete 3${}^{\prime }$ UTR assemblies. It uses a statistical model to infer change points in a gene exhibiting a sharp increase or decrease in read-coverage and employs mathematical constraints to filter false APA sites. Although rare, introns occur in 3${}^{\prime }$ UTRs and regulate gene expression [116,117]. Neither GETUTR nor IsoSCM consider intronic regions in their analysis and miss 3${}^{\prime }$ UTRs that contain introns [113].

**APAtrap** [118] uses an approach different from change-point or poly(A) peak calling (see Section 4.2.3). It extracts the known 3${}^{\prime }$ UTR from genome annotations for each gene and extends it by a pre-defined length. A sliding window is used to scan the extended region by 1bp increments to identify changes in read coverage. The location of 3${}^{\prime }$ UTR ends is determined by considering the mean read coverage in the current window, the previous window and the next window and a 3-step criterion is used to identify the precise 3${}^{\prime }$ ends. The newly identified 3${}^{\prime }$ UTRs are compared with the original genome annotation to procure novel 3${}^{\prime }$ UTRs, the 3${}^{\prime }$ end locations of which are then defined as the distal poly(A) sites. It then applies a least-squares model on read-coverage depth to identify the precise positions of poly(A) sites for each gene.

#### 4.2.3. *de novo* APA Detection in 3${}^{\prime }$ Focused Data

For every protocol listed in Table 1, bioinformatic methods were employed for data analysis. While some of them remain ad-hoc, others are available as stand-alone pipelines or packages which are discussed in this section.

The first reported **change-point model** [119] is based on a likelihood ratio test that detects any change in 3${}^{\prime }$ UTR length. It assumes the existence of two 3${}^{\prime }$ UTR isoforms in a gene, with a proximal and a distal poly(A) site. It then captures the percentage of read counts corresponding to each isoform, quantifies the expression ratio of the two isoforms across two conditions, treatment and control. The method also assumes a constant expression ratio of the two isoforms throughout the 3${}^{\prime }$ UTR and tests for changes in the expression ratio. A change in this ratio marks the 3${}^{\prime }$ UTR switching event and the site identifies as a poly(A) site. The Perl software can handle data from both RNA-seq and 3${}^{\prime }$ focused protocols and has been tested for SAPAS [63].

Different from change point models, the bioinformatic methods developed for 3${}^{\prime }$ focused RNA-seq identify poly(A) sites by peak-calling. Reads containing untemplated poly(A) sequences when compared to a reference genome are identified as 3${}^{\prime }$ ends.

**Tail Tools** [56] is a suite of tools to process and analyse the reads rich in poly(A) tails. Tail Tools measures differential gene expression, differential poly(A) tail length and differential 3${}^{\prime }$ end usage per gene. All the reads associated with each identified poly(A) peak are counted for each sample. The weitrix Bioconductor package [120] assigns a “shift score” and an associated precision weight to each gene with two or more APA sites relative to typical site usage. These scores and weights can then be used with limma [121] and topconfects [122] for differential testing. The topconfects package provides confidence bounds on the differential genes, thus provides a ranked gene list in the order of confident effect size i.e., how much shift is observed in the genes. Weitrix can handle data from both 3${}^{\prime }$ focused RNA-seq methods and from single-cell RNA-seq experiments. Along with differential poly(A) site usage, it can also find differential tail length, and introduces some exploratory features like finding components of variation in data and identify genes with excess variation (or highly variable genes, HVGs). These additional tools can also be applied to other 3${}^{\prime }$ focused RNA-seq data such as Quant-seq and 10X Genomics single-cell RNA-seq data.

**PolyA-miner** [8] creates a matrix of poly(A) sites (as rows) and samples (as columns) from 3${}^{\prime }$ focused sequencing data to apply non-negative matrix factorization which captures gene expression patterns. It first extracts all potential sample-wise poly(A) sites and pools them to construct a poly(A) library and then extensively filters out false poly(A) sites and maps the rest to their respective genes. The number of reads mapped gives the poly(A) peak count for each gene. The method accounts for all APA changes between proximal, intermediate and distal APA sites.

Application for mapping EnD-Seq data (**AppEnD**) [71] was reported along with EnD-Seq protocol but can also process data from PAS-Seq and A-Seq protocols and has the ability to automatically detect internally mis-primed A-tails, thus keeping only the true polyadenylated 3${}^{\prime }$ ends. It outputs the transcript abundance ending at each nucleotide, resulting in a positional distribution of last templated nucleotides.

Most of these tools only identify UTR-APAs. They rely on gene annotations from reference genomes in ENSEMBL which provides annotations for 3${}^{\prime }$ UTRs [123], but these are not differentiated by APA type. Independent of the reference genome annotations, mountainClimber [124] locates change points in the RNA-seq read coverage data to identify APA sites in coding and intronic regions and thus, differentiate between the two APA types.

#### 4.2.4. APA Detection in 3${}^{\prime }$ Tag-Based Single-Cell RNA-seq Data

The 3${}^{\prime }$ focussed scRNA-Seq methods such as the popular 10X Chromium encouraged the development of bioinformatic tools to resolve complexity and study APA dynamics in single-cell data, which are discussed in this section.

Modeling and Visualization of dynamics of Alternative PolyAdenylation (**MovAPA**) [125] is an R package to measure APA. It extracts poly(A) site annotations from multiple sources like PolyASite2.0, PolyA_DB3, PlantAPAdb [126], APASdb, TAPAS, APAtrap, DaPars and Cufflinks [127] to construct a library that stores expression levels, annotation, and sample information of poly(A) sites from different samples which is then used for the downstream analysis. While movAPA relies on prior poly(A) annotations, the following tools identify poly(A) peaks or compute differential APA usage *de novo*.

**BATBayes** [92] uses a statistical framework to compare variability in 3${}^{\prime }$ UTR isoform usage in homogeneous cell populations from BAT-seq data. The analysis identifies poly(A) sites by UMI counting and only considers the two most abundant 3${}^{\prime }$ UTR isoforms for each gene.

**scAPA** [128] is an R-script that combines various toolkits such as Samtools [129], Bedtools [130], Homer, UMI_tools [131], etc. for their analysis. It uses Homer to detect poly(A) site by peak-calling and uses *mclust* to separate overlapping peaks based on a Gaussian mixture model. It employs *featureCounts* [132] to quantify peak usage in each cell-type cluster and performs a $\chi^{2}$-test to detect dynamic APA events.

**Sierra** [81] applies the DEXSeq package [133], originally designed to detect differential exon usage in bulk RNA-Seq data, to APA usage in pseudo-bulk samples. As DEXSeq performs tests based on the negative binomial distribution, this method takes biological variation into account, which many other methods fail to do.

**scAPAtrap** [82] employs peak-calling to detect potential poly(A) sites and integrates poly(A) read anchoring where reads with A/T stretches are used to determine the precise locations of the poly(A) sites, which other methods like scAPA and Sierra fail to do. It also splits the overlapping peaks into smaller peaks and then employs the movAPA package to compute APA.

**scDAPA** [134] computes the APA difference between samples or between cell-types within the same sample. It doesn’t call poly(A) sites, instead, it employs a histogram-based approach to divide the reads in 3${}^{\prime }$ ends into bins of the same width and computes a difference in the percentage of reads in each bin for a gene across two conditions. A Wilcoxon rank-sum test measures the significance of the differential APA usage in these bins.

## 5. The Repertoire of Cancer Biomarkers

The seminal study by Mayr and Sharp (2009) first showed the association of APA with cancer. Since that time, APA has been reported in multiple studies of cancer proliferation and transformation, as extensively reviewed by Gruber and Zavolan (2019) [39]. These APA genes have the potential to be used as prognostic markers in predicting cancer progression, risk stratification and even for developing personalised therapies [16,22,34,83,135,136,137,138,139].

Current prognostic tests rely on gene expression profiles [140,141]. But these may be improved by incorporating APA. Several APA genes have been proposed as novel prognostic biomarkers and some examples are shown in Table 4. These gene expression and APA signatures could be combined with drug-sensitivity data, and clinical covariates such as patient age, survival time, tumour stage, location and size to build a multivariate regression model [137,138]. For example, a recent study used linear regression model to connect APA events and drug sensitivity with clinical relevance, supporting their utility as biomarkers [137].

A 17-gene 3${}^{\prime }$ UTR-based classifier was reported that divided patients into high and low risk groups, predicting risk in patients with triple-negative breast cancer (TNBC) significantly better than the classical clinicopathological risk [138]. The prognostic model in this study reported 10 APA genes that undergo 3${}^{\prime }$ UTR shortening and were associated with poor prognosis. It also reported 7 APA genes that undergo 3${}^{\prime }$ UTR lengthening and were associated with poor prognosis showing that APA-mediated gene regulation is more complicated than was first thought. In an important caveat, this study found the SMAD6 gene to be associated with poor prognosis in TNBC patients but that it favours survival in lung cancer patients, indicating that the APA events are tumour-dependent. The expression of APA genes detected by single-cell RNA-seq are now being shown to correlate with clinical outcomes of early-stage breast cancer in a single-cell data [83]. They report 53 cancer cell-specific APA genes with a distinct pattern of 3${}^{\prime }$ UTR shortening and an immune-specific APA signature with possible clinical utility in early stage breast cancer. However, of the many potential clinically relevant APA genes that have been reported, most have yet to be independently clinically validated.

In a disease setting like TNBC, which is highly aggressive and has a high recurrence rate, the lack of hormone receptors means the targeted therapies are not applicable. As a result, patients are treated with conventional radiotherapy or chemotherapy [143]. Better treatment methods are required. APA markers or the mechanism that cause APA could be used as targets for development of novel treatment therapies [144,145].

Based on current literature, APA appears to be associated with tumorigenicity in all cancer patients. The time is therefore ripe to take these smaller scale research findings into larger cohort studies to mine the full potential of APA as novel cancer biomarkers.

## 6. Conclusions

APA is an established mechanism for the generation of transcriptome diversity that impacts basic cellular functions, cancer proliferation and transformation and ultimately controls cellular fate. The development of bespoke RNA-seq technologies combined with bioinformatic methods and curated databases have paved the way for the potential of APA as cancer biomarkers to be tested at scale. These APA markers, if combined with standard prognostic measures such as gene expression and clinical covariates may contribute toward development of novel diagnostic tests and may facilitate personalised cancer therapies. 

## Figures and Tables

**Table 1:** 3${}^{\prime }$ focused RNA-sequencing approaches suitable for APA detection and characterization.

Name	Key Points	Typical Input	Sequence Target
PAIso-seq [52]	PacBio based method to capture poly(A) site, length, splicing, expression, PacBio is costly for the read coverage obtained, Low coverage	100 ng total RNA	Full length mRNA, Poly(A) tail included
Oxford Nanopore- Direct RNA sequencing [53]	The Nanopore instrument is capable of full-length direct RNA seq, tail lengths can also be extracted. Low coverage	500 ng poly(A)+ selected RNA	Full length mRNA, Poly(A) tail included
TAIL-seq [54]	rRNA depletion and 3${}^{\prime }$ adaptor ligation, asymmetric paired end sequencing to determine tail length	∼100 g total RNA	Poly(A) tail length, Poly(A) site
mTAIL-seq [55]	3${}^{\prime }$ oligo(dT) splinted ligation approach to TAIL-seq, reduced input RNA required. Paired-end sequencing.	1–5 g total RNA	Poly(A) tail length, Poly(A) site
PAT-seq [56]	Single end read approach, 3${}^{\prime }$ tagging by oligo templated RNA end extension	1 g total RNA	Poly(A) tail length, Poly(A) site
PAL-seq [57]	Requires non-standard use of an Illumina instrument for tail length measurement by biotinylated dTTP incorporation. 3${}^{\prime }$ end capture by splinted ligation	1–50 g total RNA	Poly(A) tail length, Poly(A) site
Poly(A) seq [58]	Poly(A)+ RNA is captured with oligo(dT) conjugated magnetic beads, then 3${}^{\prime }$ adaptors ligated 300 bp single end read. Samples sequenced on the Illumina NextSeq 500, 2 colour sequencing instrument	5.1 g total RNA	Poly(A) tail length, Poly(A) site
TED-Seq [59]	3${}^{\prime }$ adaptor ligation to Poly(A)+ RNA. Tail length is inferred from the size of the templated sequence after precise library size selection	100 ng poly(A)+ RNA	Poly(A) tail length, Poly(A) site
3P-seq [4]	Poly(A) tail removed by RNase H. Sequenced from the 3${}^{\prime }$ end to determine site usage, adaptor addition by ligation to avoid internal priming	30 g total RNA	Poly(A) site
2P-seq [60]	Poly(A) site detection by anchored oligo(dT) priming, sequencing from start of poly(A) tail in reverse	15 g total RNA	Poly(A) site
3${}^{\prime }$-seq [30]	Poly(A) site detection by anchored oligo(dT) priming. Unique approach to fragmentation by rate limited nick translation of double stranded cDNA	2 g DNase treated RNA	Poly(A) site
3${}^{\prime }$READS+ [37]	Poly(A) tail is trimmed by RNase H, 3${}^{\prime }$ adapter ligation	0.1–15 g total RNA	Poly(A) site
3PC [61]	Anchored oligo(dT) primer to detect poly(A) site, 5${}^{\prime }$ adaptor addition by circular ligation	100 g total RNA	Poly(A) site
3${}^{\prime }$T-fill [62]	Anchored oligo(dT) primer to detect poly(A) site, sequenced from 3${}^{\prime }$ end. 3${}^{\prime }$T-fill reaction - dA homopolymer region at 3${}^{\prime }$ end filled with dTTPs on Illumina cBot cluster station before sequencing	0.5–10 g total RNA	Poly(A) site
SAPAS [63]	Anchored oligo(dT) primer to detect poly(A) site, 5${}^{\prime }$ adaptor addition by template switching	10 g total RNA	Poly(A) site
PAS-seq [5]	Anchored oligo(dT) primer to detect poly(A) site, template switching 5${}^{\prime }$ adaptor addition	0.5–1 g poly(A)+ selected RNA	Poly(A) site
IVT-SAPAS [64]	*in vitro* transcription based amplification of cDNA for low input samples, poly(A) site detection by anchored oligo(dT) annealing	200 ng total RNA	Poly(A) site
PAPERCLIP [65]	RNA crosslinked, partially digested, and 3${}^{\prime }$ ends immunoprecipitated via Poly(A) Binding protein, addresses internal priming issues, uses anchored oligo(dT) annealing for end detection	NA, starting material is tissue/cells	Poly(A) site
MACE [66]	GenXPro commercial kit, barcodes transcripts with UMIs to deal with PCR duplication	0.05 ng total RNA	Poly(A) site
Quant-Seq [67]	Lexogen commercial kit, oligo(dT) annealing to detect 3${}^{\prime }$ ends, random forward priming of 2nd strand cDNA adds 5${}^{\prime }$ adaptor	0.5–500 ng total RNA	Poly(A) site
MAPS [68]	3${}^{\prime }$ end detection by anchored oligo(dT) priming, 5${}^{\prime }$ adaptor addition by random forward priming of 2nd stand cDNA	1 g total RNA	Poly(A) site
TM3${}^{\prime }$seq [69]	Fragmentation and 5${}^{\prime }$ adaptor addition combined in a single step. 3${}^{\prime }$ end detected via annealing of oligo(dT) primer	200 ng total RNA	Poly(A) site
PAC-seq [70]	Click-chemistry approach to fragmentation and 5${}^{\prime }$ adaptor addition via reverse transcription termination by 3-azido-nucleotides. 3${}^{\prime }$ end detected by oligo(dT) annealing	0.125–4 g total RNA	Poly(A) site
EnD-Seq [71]	Targeted sequencing approach to 3${}^{\prime }$ end detection, 3${}^{\prime }$ adaptor ligation to total RNA, gene specific multiplex PCR of cDNA	1.5 g total RNA	Poly(A) site, non-Poly(A) 3${}^{\prime }$ ends

**Table 2:** Single cell RNA-sequencing approaches suitable for APA detection and characterization.

Name	Overview	Scale
CEL-seq [86]	3${}^{\prime }$ ends enriched by anchored oligo(dT) annealing including T7 promotor. cDNA amplified by *in vitro* transcription (IVT), amplified RNA fragmented and ligated to adaptor.	Manually isolated single cells
CEL-seq2 [87,88]	Application of CEL-seq to high throughput sequencing, UMI’s added to reverse transcription oligo	Automated microfluidic sorting via Fluidigm C1 into wells
MARS-seq 2.0 [89]	3${}^{\prime }$ end enrichment by anchored oligo(dT) annealing, included T7 promotor. cDNA amplified via IVT	384-well plate, FACS sorting
InDrop [80]	Application of CEL-seq to droplet-based sequencing for higher throughput	Droplet sequencing, inDrop system, 1CellBio
Drop-seq [90]	3${}^{\prime }$ enrichment by oligo(dT) annealing RT, full length cDNA 5${}^{\prime }$ labelled by template switching, oligo’s with common barcode bound to beads, and separated into droplets. library prepared by Illlumina Nextera XT DNA library prep kit	Droplet sequencing, custom instrument
10X Chromium [85]	3${}^{\prime }$ enrichment by anchored oligo(dT) annealing, oligo’s with common barcode bound to beads, and separated into droplets; library preparation with commercial kit GemCode Single-Cell 3${}^{\prime }$ Gel Bead and library kit (now Chromium 10X)	Droplet sequencing, 10X genomics instrument
SCRB-seq [91]	3${}^{\prime }$ enrichment by anchored oligo(dT) primer, template switching reaction for full length cDNA, library prepared by Illlumina Nextera XT DNA library prep kit	384-well plate, FACS sorting
MAPS-seq [84]	3${}^{\prime }$ ends enriched by biotinylated oligo(dT) annealing, RNA transcripts pulled down and samples pooled together using magnetic beads before RT. Full length cDNA 5${}^{\prime }$ adaptor added via template switching, library prepared by Illlumina Nextera XT DNA library prep kit	96-well plate, FACS sorting
BATSeq [92]	Method specifically developed to detect APA. 3${}^{\prime }$ ends enriched by oligo(dT) annealing. 2nd strand cDNA IVT amplified	FACS sorting

**Table 3:** Bioinformatic databases for 3${}^{\prime }$ UTR and APA storage and retrieval.

Database	Primary Data Collection	Organism	Last Updated	URL
UTRdb [93]	5${}^{\prime }$ and 3${}^{\prime }$ UTR regions in EMBL/GenBank records	human, rodent, vertebrate, plant and fungi	2010	http://utrdb.ba.itb.cnr.it/
PACdb [95]	cDNA/ESTs	human, mouse, rat, dog, chicken, zebrafish, fugu, fruit fly, mosquito, nematode, Arabidopsis thaliana, rice and baker’s yeast	inaccessible	http://harlequin.jax.org/pacdb/
PolyA_DB [72,94,106]	aligned cDNA/ESTs	human, mouse, rat, chicken and zebrafish	2018	http://polya-db.org/v3/
GENCODE Poly (A) site track [100,101]	cDNA/ESTs	human	2021	https://genome.ucsc.edu/cgi-bin/hgTrackUi?db=hg19&g=wgEncodeGencodeV19
APADB [97]	MACE-Seq	human, mouse and chicken	2014	http://tools.genxpro.net/apadb/
APASdb [96]	SAPAS	human (22 normal and cancer tissues), mouse, zebrafish and some lancelet samples	inaccessible	http://mosas.sysu.edu.cn/utr
TC3A [99]	RNA-seq in TCGA	32 human cancer types	inaccessible	http://tc3a.org/
APAatlas [27]	RNA-seq in GTEx project	>50 human normal tissue	2020	https://hanlab.uth.edu/apa/
PolyASite [98]	3${}^{\prime }$-Seq, 3${}^{\prime }$READS, DRS, QuantSeq_REV, SAPAS, PAPERCLIP, PolyA-seq, PAS-Seq, A-seq, 3P-Seq, DRS, 2P-Seq, PAT-seq	human, mouse and worm	2020	https://polyasite.unibas.ch/

**Table 4:** APA genes as potential cancer biomarkers.

Cancer	Gene Markers	Signature APA	Physiological Effects	Molecular Role
Breast	PRELID1	Shortening of 3${}^{\prime }$ UTR	increased protein expression	mitochondrial ROS signalling [139]
Breast	SNX3, YME1L1D, USP9X	Shortening of 3${}^{\prime }$ UTR	increased protein levels in short isoform	EGF signalling [22]
adult T-cell lymphoma, large B-cell lymphoma, stomach adenocarcinoma	PD-L1 gene (CD274)	Shortening of 3${}^{\prime }$ UTR	PD-1/PD-L1-mediated immune escape in cancer development; structural variants (SVs) disrupt 3${}^{\prime }$ regulatory region of PDL1	T-cell modulator; PDCD1-mediated inhibitory pathway [136]
Colorectal cancer	IGF2BP1/IMP-1	Shortening of 3${}^{\prime }$ UTR	increased protein levels; increased oncogenic transformation	Modulates pathogenesis [142]
TNBC, lung, esophageal, bladder, leukemia, ovarian	N4BP2L2, WDHD1, ZER1, ADGRL2, PRSS12, NPL, SIK3, SYNGR1, SCL2A3, UBE2G2	Shortening of 3${}^{\prime }$ UTR	unfavourable prognosis	All are related to cancer development: cell cycle regulator and is involved in PI3K/Akt pathway; tumour antigen [138]
TNBC	PPIC, ZCCHC14, RTN1, PRCK8, CLIC2, CXCL8, SMAD6	Lengthening of 3${}^{\prime }$ UTR	poor prognosis; response elements (MREs) in the lengthened 3${}^{\prime }$ UTR leads to homologous gene repression and competing endogenous RNA (ceRNA) resulting in cancer progression; more miRNA binding sites	TGF-βpathway; autocrine NF-ƙB/IL-8 (CXCL8) pathway responsible for cell migration; aberrant pathways and cancer progression [138]
TNBC (MB-231)	Caspase 6, DFFA (ICAD), DFFB (CAD), PARP1	Lengthening of 3${}^{\prime }$ UTR	escape of apoptosis	Caspase pathway [63]
TNBC (MB-231)	cyclin D1, D2	Shortening of 3${}^{\prime }$ UTR	promote cell cycling	Mitotic cell cycle; APC [63]
